# Diffuse Maculopapular Rash: An Uncommon Cutaneous Presentation of Secondary Syphilis

**DOI:** 10.7759/cureus.74087

**Published:** 2024-11-20

**Authors:** Landon Hobbs, Redina Bardhi, Priyanka Shah, Ali Rajabi-Estarabadi, Asfa Akhtar

**Affiliations:** 1 Department of Dermatology, Cleveland Clinic Florida, Weston, USA

**Keywords:** cutaneous syphilis, dermatology, dermatopathology, infectious venereal disease, public health and safety, secondary syphilis, sexually transmitted infection (sti), suspected drug eruption, syphilis rash

## Abstract

Syphilis is a sexually acquired disease that can affect multiple organ systems. Secondary syphilis can cause a wide range of skin manifestations, leading to misdiagnoses. Herein, we present a patient who developed a diffuse maculopapular rash concerning for a drug reaction leading to a dermatology consult. The patient was found to have secondary syphilis confirmed with immunohistochemical staining on skin biopsy tissue and a positive rapid plasma reagin. Appropriate management with benzathine penicillin G led to significant improvement in the rash. Given the increasing incidence of syphilis, increased awareness of the disease is imperative so that it can be considered in all patients given the highly variable presentation and likely confusion, at least initially, with drug reaction with eosinophilia and systemic symptoms syndrome and other drug eruptions.

## Introduction

Syphilis is a highly contagious sexually acquired disease caused by the spirochete *Treponema pallidum* that can affect multiple organ systems. Secondary syphilis is referred to as "the great imitator/mimicker" due to its wide range of skin manifestations, as well as systemic symptoms [1-4]. It is typically preceded by a nontender genital chancre (primary syphilis) and can progress to tertiary syphilis if left untreated [1-3]. Given the variable clinical manifestations, it can be confused with many different diseases. The incidence of syphilis is increasing rapidly, thus making it a greater public health issue [1-4]. Herein, we present a case of a 45-year-old man with secondary syphilis that was masquerading as a drug rash in a patient with no recollection of a primary syphilis chancre. It highlights the importance of the inclusion of syphilis in the differential diagnosis given the increasing prevalence, and that can lead to multiple sequelae if left untreated. This case was previously presented as a meeting abstract at the 2022 Florida Academy of Dermatology Annual Meeting on July 24, 2022.

## Case presentation

**Figure 1:**
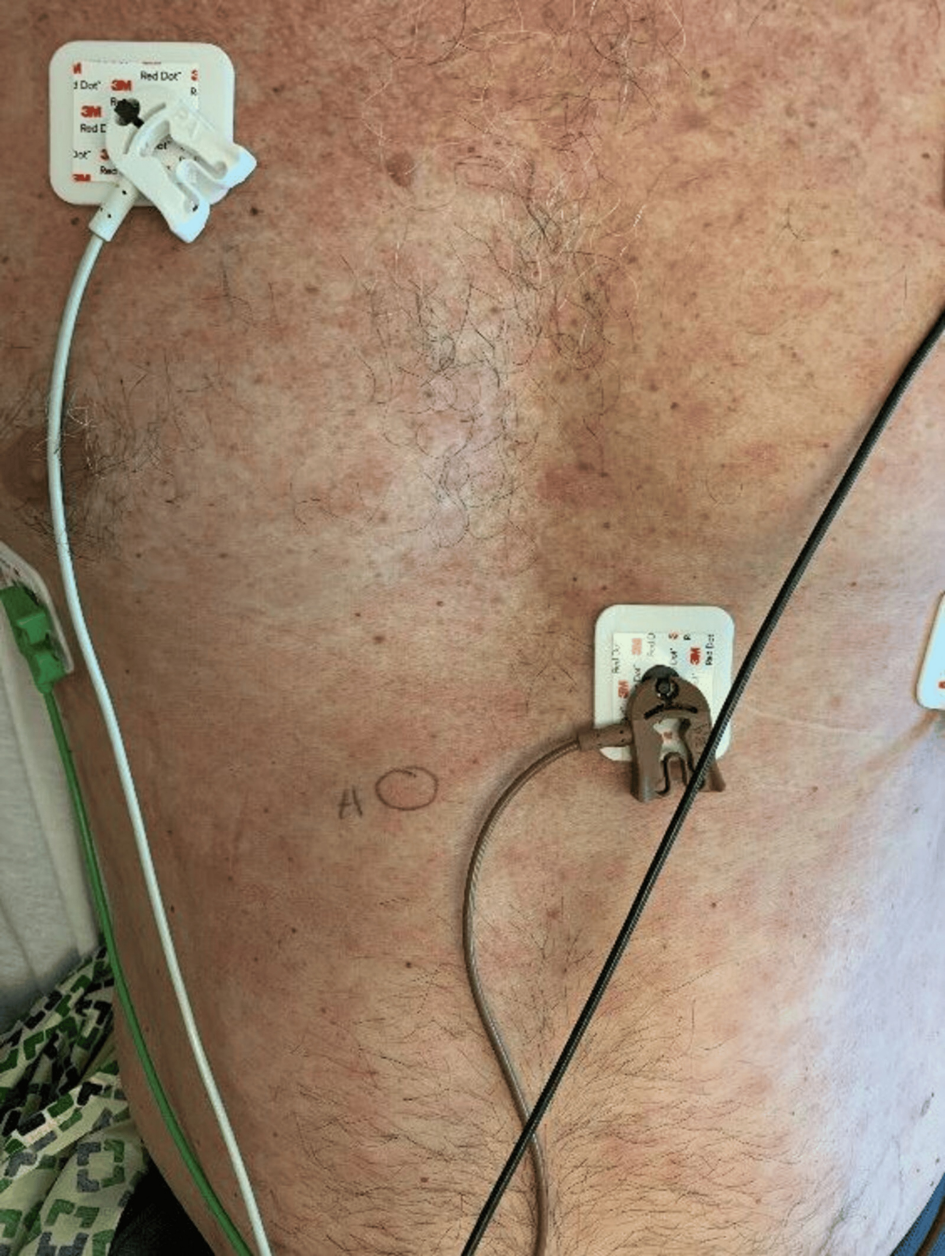
Clinical photograph of patient's chest and abdomen at the time of presentation, where the patient had a widespread eruption of erythematous maculopapular rash involving the abdomen and chest

**Figure 2:**
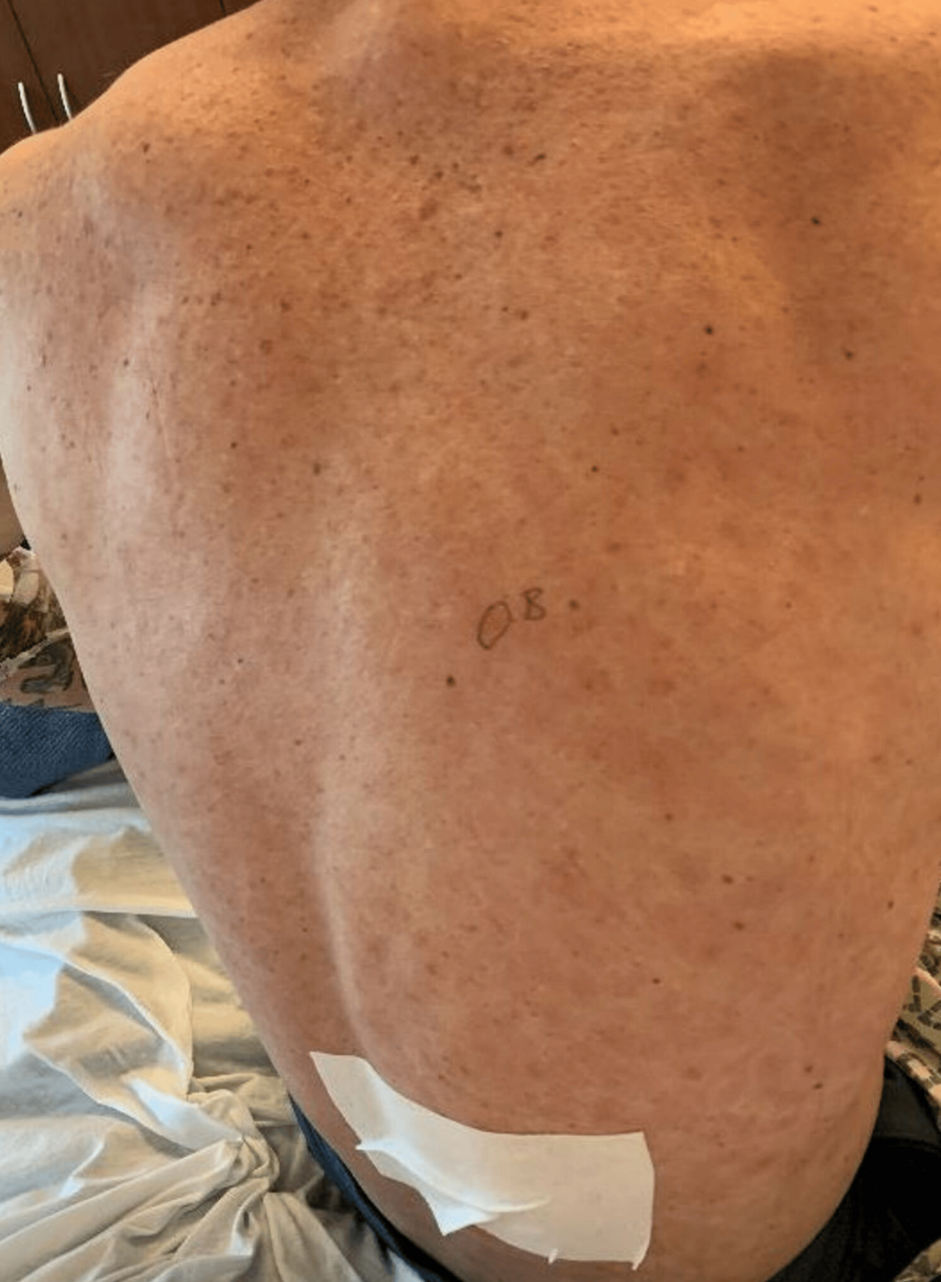
Clinical photograph of the patient's back, displaying erythematous maculopapular rash at time of presentation

**Figure 3:**
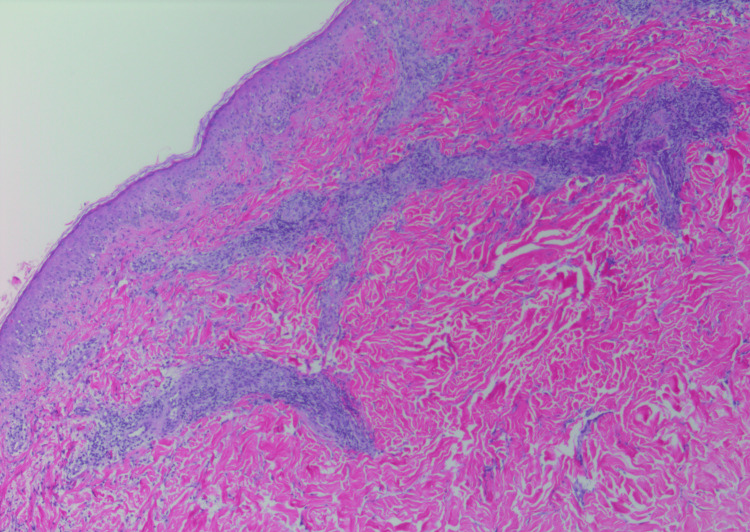
Histopathology photograph, displaying superficial and mid-to-deep dermal perivascular inflammatory cell infiltrate with rare plasma cells with vacuolar interface changes of the overlying epidermis associated with necrotic keratinocytes (hematoxylin and eosin, 40×)

**Figure 4:**
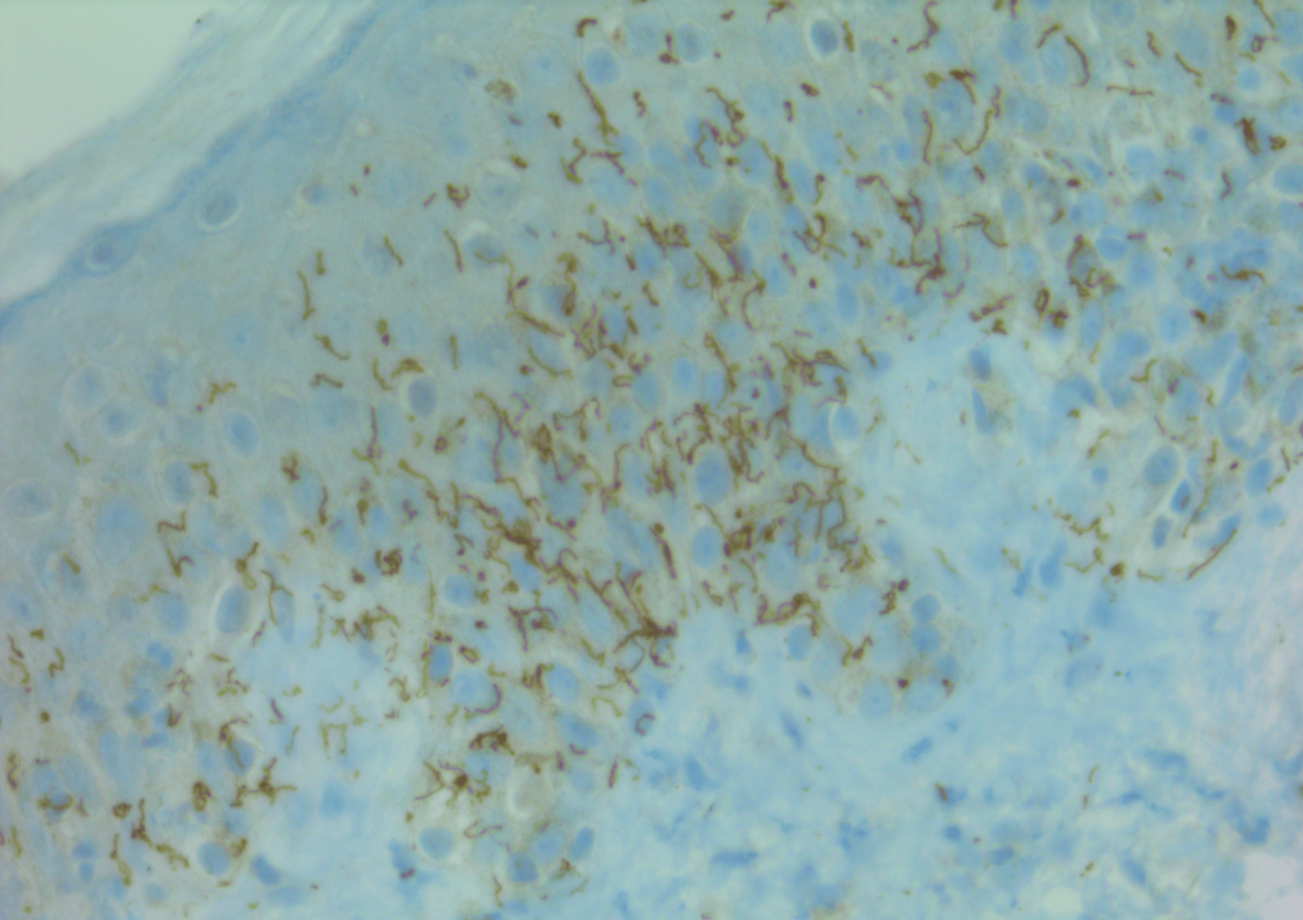
Immunoperoxidase highlighting T. pallidum in the epidermis. Skin biopsy staining positive for spirochetes with immunohistochemistry (T. pallidum antibody, 400×)

A 45-year-old man with a history of hypertension, hypercholesterolemia, and type I diabetes mellitus was admitted with a four-week history of abdominal pain and a two-week history of a diffuse macular rash. He reported no fevers, weight loss, fatigue, myalgias, arthralgias, or photosensitivity. Initial laboratory testing was significant for transaminitis, hyperbilirubinemia, positive antinuclear antibodies, and elevated inflammatory markers (Table 1). The rash spread from his trunk to both hands and wrists during admission. Dermatology was consulted for drug eruption vs. drug reaction with eosinophilia and systemic symptoms (DRESS) syndrome. Physical examination showed a widespread nonpruritic maculopapular, or morbilliform, eruption characterized by pink papules and macules extending to the palms and soles (Figures 1, 2). Skin biopsy was performed to further evaluate the rash and showed superficial and mid-to-deep dermal perivascular chronic inflammatory cell infiltrate with rare plasma cells with vacuolar interface changes of the overlying epidermis associated with necrotic keratinocytes (Figure 3). Given the presence of plasma cells, an immunoperoxidase stain for *T. pallidum* was performed, and spirochetes within the epidermis were revealed (Figure 4). Further investigation revealed a reactive syphilis panel, including reactive *T. pallidum* antibodies in the serum and a reactive rapid plasma reagin (RPR) with a titer of 1:8, as well as positive staining for *T. pallidum* in a previously obtained liver biopsy confirming liver involvement by syphilis. Upon discussion of the diagnosis, the patient stated that he recently discovered that his partner cheated on him. The patient was diagnosed with secondary syphilis and subsequently treated with a single dose of 2.4 million units of benzathine penicillin G. Four weeks later, at a follow-up visit, his rash had significantly improved.

**Table 1:** Patient's laboratory results Abs: antibodies; ALT: alanine transaminase; ANA: antinuclear antibodies; AST: aspartate aminotransferase; CRP: C-reactive protein; ESR: erythrocyte sedimentation rate; Hb: hemoglobin level; RBC: red blood cell count; RPR: rapid plasma reagin; WBC: white blood cell count

Name of laboratory test (unit)	Value	Normal range
WBC (k/uL)	6.55	3.7-11.0
RBC (m/uL)	4.15	4.2-6.0
Hb (g/dL)	13.1	13.0-17.0
Platelet count (k/uL)	290	150-400
Absolute eosinophils (k/uL)	0.05	<0.46
ESR (mm/hour)	23	0-15
CRP (mg/dL)	1.4	<0.5
Transferrin (mg/dL)	156	176-315
Bilirubin, total (mg/dL)	2.9	0.2-1.3
Bilirubin, direct (mg/dL)	1.7	<0.2
Alkaline phosphatase (U/L)	385	38-113
AST (U/L)	100	14-20
ALT (U/L)	247	10-54
Lipase (U/L)	13	16-61
*T. pallidum* Abs	Reactive	Nonreactive
RPR, qualitative	Reactive	Nonreactive
RPR, quantitative	1:8	<1:1
ANA with titer	1:160	<1:80

## Discussion

Syphilis is a highly contagious sexually transmitted infection that can progress through four stages, including primary, secondary, and tertiary syphilis, as well as a latent period of variable length before the onset of tertiary syphilis [1-4]. Primary syphilis classically presents as a solitary nontender genital chancre 10-90 days after the initial infection, but patients can also have nongenital chancres. Secondary syphilis is frequently referred to as "the great imitator/mimicker" due to its wide range of skin manifestations, including macular or papular rashes that can involve the palms and soles, and alopecia, as well as systemic symptoms such as lymphadenopathy, hepatitis, hepatosplenomegaly, myalgias, and neurologic deficits that result from hematogenous dissemination of the spirochete [1-3]. The lesions and symptoms of both primary and secondary syphilis can resolve without treatment but, when left untreated, can progress to secondary and tertiary syphilis, respectively. Afterward, the patient enters a latent phase of variable duration in which there are no clinical signs of the disease, but it can be detected with serologic testing. Tertiary syphilis is characterized by cardiovascular syphilis, neurosyphilis, or late benign syphilis [1-3].

DRESS syndrome typically presents as a morbilliform rash with systemic symptoms, including fever, edema, and lymphadenopathy, as well as evidence of internal organ involvement such as transaminitis or abnormal renal function on laboratory testing. It is triggered by medications such as antiepileptics, antibiotics, and nonsteroidal anti-inflammatory drugs [5]. Our patient had a similar rash and transaminitis but lacked fever, eosinophilia, edema, and lymphadenopathy. Our patient was found to have liver involvement by syphilis, confirmed by immunohistochemical staining, which was the underlying cause of the patient's transaminitis rather than DRESS syndrome. Liver involvement by *T. pallidum*, also known as syphilitic hepatitis, has been previously reported in patients with transaminitis and abdominal symptoms, like our patient, that were initially worked up for DRESS syndrome [6]. The most common clinical manifestations of syphilitic hepatitis include rash, fatigue, icterus, fever, weight loss, and abdominal pain [7]. Although our patient had a biopsy confirming syphilitic hepatitis, it has been proposed that syphilitic hepatitis can be diagnosed without a biopsy in patients with transaminitis, positive syphilis serology, exclusion of other causes of liver disease, and liver enzymes returning to normal with the treatment of syphilis [6,7]. Drug eruptions commonly present as a morbilliform rash that spreads centrifugally, from trunk to extremities, in a bilateral and symmetrical pattern after exposure to the triggering medication. The most commonly implicated medications include beta-lactams, sulfonamides, and antiepileptic medications [8]. The rash in our patient was morbilliform and spread centrifugally. Given the potential for significant overlap in clinical presentation between DRESS syndrome, secondary syphilis, and a morbilliform drug eruption, a thorough clinical history, specifically an accurate medication history and timeline, is necessary to differentiate these conditions. Additional testing, including biopsy and serologic testing, may be required to distinguish these entities. The underlying cause and subsequent treatment are different, so making the correct diagnosis is imperative. Our patient did not take any of the commonly associated medications for DRESS or morbilliform drug eruption.

The diagnosis of syphilis is made with positive serologic testing, including RPR, venereal disease research laboratory, fluorescent treponemal antibody absorption, and treponema pallidum particle agglutination. Histopathology findings typically include irregular acanthosis with elongated rete ridges, endothelial swelling, a vacuolar pattern with lymphocytic infiltration, and the presence of plasma cells [1-3]. Histopathologic evaluation of the skin biopsy of our patient showed the vacuolar lymphocytic infiltrate with rare plasma cells concerning syphilis, with subsequent immunohistochemistry and RPR confirming the diagnosis. Treatment for primary, secondary, and early latent syphilis is a single dose of 2.4 million units of intramuscular benzathine penicillin G; tertiary syphilis requires weekly intramuscular 2.4 million units of penicillin for three weeks. Alternative therapies include 14 days of either 100 mg oral doxycycline twice daily or 1-2 g intramuscular/intravenous ceftriaxone [1-4]. Our patient's rash significantly improved with intramuscular benzathine penicillin.

The incidence of syphilis in adults is increasing at a rapid rate [1-4,9]. The incidence of syphilis in the United States reached a historic low in 2000; however, since 2001, there has been a steady increase [2]. Data from the Centers for Disease Control and Prevention show that the incidence of syphilis among adults in the United States increased by 38% from 2008 to 2012 and by 80% between 2018 and 2022 [1]. A 10-fold increase in cases was reported in the United Kingdom over a 15-year period [3]. Worldwide prevalence was found to increase by 60% from 1990 to 2019 [4]. In a four-year hospital-based retrospective study in India, syphilis patients made up 4.36% of patients seen in the venereal disease clinic, but the annual incidence increased from 4.73% to 5.27% from the beginning to the end of the study. Of the 137 syphilis patients, 94.16% were found to have latent stage syphilis on routine screening, whereas only 1.45% presented with a primary chancre and 4.37% presented with secondary syphilis. The percentage of patients with latent syphilis was found to be increased compared to prior studies in India, suggesting that the increasing proportion of latent syphilis may be leading to underestimation of the rising incidence of syphilis. It was hypothesized that the increase in latent syphilis might be secondary to a long incubation period, inadvertent use of antibiotics for unrelated conditions partially treating syphilis, or patients with asymptomatic primary or secondary syphilis skipping treatment [9].

## Conclusions

Secondary syphilis can be a mimicker of many different diseases and can lead to significant complications if left untreated. It can affect multiple organs, as noted by the involvement of our patient's skin and liver, and thus can cause a lab abnormality of the organ it affects. Given the rapidly increasing incidence of syphilis, increased awareness and recognition of the disease is imperative to facilitate early treatment to avoid sequelae. Secondary syphilis should be considered in all patients presenting with a morbilliform rash, given the overlapping features and possible confusion, at least initially, with DRESS syndrome and drug eruptions in patients who recently started a new medication.

## References

[1] Tudor ME, Al Aboud AM, Leslie SW, Gossman W (2023). Syphilis. https://www.ncbi.nlm.nih.gov/books/NBK534780/.

[2] Cohen SE, Klausner JD, Engelman J, Philip S (2013). Syphilis in the modern era: an update for physicians. Infect Dis Clin North Am.

[3] Nyatsanza F, Tipple C (2016). Syphilis: presentations in general medicine. Clin Med (Lond).

[4] Tao YT, Gao TY, Li HY (2023). Global, regional, and national trends of syphilis from 1990 to 2019: the 2019 global burden of disease study. BMC Public Health.

[5] Calle AM, Aguirre N, Ardila JC, Cardona Villa R (2023). DRESS syndrome: a literature review and treatment algorithm. World Allergy Organ J.

[6] Alemam A, Ata S, Shaikh D, Leuzzi B, Makker J (2021). Syphilitic hepatitis: a rare cause of acute liver injury. Cureus.

[7] Huang J, Lin S, Wan B, Zhu Y (2018). A systematic literature review of syphilitic hepatitis in adults. J Clin Transl Hepatol.

[8] Crisafulli G, Franceschini F, Caimmi S (2019). Mild cutaneous reactions to drugs. Acta Biomed.

[9] Kaur R, Gupta S, Sarangal R, Chopra D, Singh H (2023). Are we moving from symptomatic to asymptomatic syphilis: a retrospective analysis. Indian J Sex Transm Dis AIDS.

